# Causal effects and functional mechanisms of the Internet on residents' physical fitness-An empirical analysis based on China family panel survey

**DOI:** 10.3389/fpubh.2022.1111987

**Published:** 2023-01-13

**Authors:** Long Zhang, Chuntian Lu, Cuixia Yi, Zhipeng Liu, Yuhua Zeng

**Affiliations:** ^1^Department of Sociology, Xi'an Jiaotong University, Xi'an, China; ^2^Department of Physical Education, Xi'an University of Technology, Xi'an, China; ^3^Physical Education School, Shaanxi Normal University, Xi'an, China; ^4^School of Economics and Management, Shanghai University of Sport, Shanghai, China

**Keywords:** Internet, health, physical fitness, causal effect, mediation effect

## Abstract

**Introduction:**

Physical fitness is an essential part of a healthy lifestyle that concerns the overall health of the nation. Research on the relationship between the Internet and physical fitness has long been caught in the dilemma of “media mobilization” and “media suppression,” and previous studies have rarely examined the causal relationship and functional mechanism.

**Methods:**

This study selected the data of 23,989 samples successfully followed in all three surveys of the China Family Panel Survey (CFPS) from 2014 to 2018 to explore the correlation and causal inference between the Internet and physical fitness by using the Time Fixed Effects Model and cross-lagged models, respectively; meanwhile, the data of 24,687 samples in CFPS 2020 to examine the functional mechanism of the Internet's effect on residents' physical fitness behavior by the KHB method.

**Results:**

We obtained three valuable conclusions as follows: First, there is a significant correlation between the Internet and physical fitness behavior. Second, the Internet use is the cause for the increase in fitness frequency, and there is a rival relationship between Internet duration and fitness time. Third, under regular prevention and control of the COVID-19 epidemic, social capital and health risk perceptions are the functional mechanisms of the Internet influencing fitness behavior, and the mediating effect of psychological health risk perceptions is higher than that of social capital.

**Discussion:**

It's necessary to create an intelligent, informative, and digital sports public service system by enriching and optimizing sports media and facilitating the Internet to serve residents' physical fitness better. The new concept of “Internet plus Fitness” will be of great significance in the implementation of the “Healthy China Initiative.”

## Introduction

Since the outbreak of COVID-19, due to physical isolation, vaccination, and other control measures, China has accomplished a remarkable achievement in the fight against the epidemic which has maximally protected the lives and health of the residents. However, the continuous mutation of the virus has caused the outbreak to repeatedly rise in the country. The negative impact of isolation on physical and mental health is increasingly prominent, despite the fact that it remains a important way of blocking the spread of the epidemic. Given this context, promoting active physical fitness by establishing the concept of “everyone is the first responsible for his or her own health” (1) should be one of the major contents of the regular prevention and control of the epidemic.

Among the current studies, the factors influencing residents' physical fitness behavior include macro social environment factors such as sports policy and system, public sports funding, service system, and public space supply (2–4), as well as micro-social hierarchy factors such as income, education and occupational status (5, 6), and also individual factors such as perceptions, attitudes and behavioral motivations toward physical fitness (7).

The Internet has played an essential role in residents' fitness behavior during the home quarantine of the epidemic. On one hand, while physical isolation has to some extent weakened the social environment and hierarchical advantages of sports, it has strengthened the impact of the Internet on residents' physical fitness behavior (8–10). On the other hand, Physical fitness strategies promoted by the Internet are undoubtedly the most effective way to address health risks and defend against crises (11). According to a survey among 21,118 residents conducted by the Zhejiang Provincial Sports Bureau on the status of physical fitness during the epidemic, 81.54% participated in physical exercise, which is a 10.23% increase from before the epidemic. 40.48% of residents engaged in home fitness 3 times a week, and 70.03% of them exercised for more than 15 min. Most residents reckoned that physical exercise contributes a lot to enhancing immune ability, promoting confidence in defending epidemics, and conquering psychological fears (12). With the regular prevention and control of the epidemic, what will be the relationship between Internet and residents' physical fitness behavior? Will the online medium play a sustainable role in elevating the physical fitness level of the population? The answer is of great practical importance in regular prevention and control of the epidemic and the protection of the physical and psychological health of the residents.

Previous studies focusing on the relationship between the Internet and residents' physical fitness behaviors have achieved some results, but have also been challenged. Firstly, research on the influence of Internet on physical fitness or sports participation has developed into two opposing theoretical perspectives which are “media mobilization” (13–15) and “media suppression” (16–18). Scholars holding the “media mobilization” argue that the Internet has shaped the social environment of sports, extended the ways of sports participation (19–21), consolidated and broadened the ways of accumulating sports social capital (22, 23), and thus influenced the residents' perceptions, attitudes and behaviors toward the value of physical fitness. Researchers holding the “media suppression” promote that the increase in time spent on the Internet comes at the cost of less time spent on physical fitness (14). The residents' physical fitness behavior is influenced by various factors such as age, gender, education level, urban and rural areas, and social class. The regression estimation method of traditional cross-sectional data pays inadequate attention to the endogeneity issue, resulting in ambiguous causal relationship between the Internet and physical fitness behaviors, and it is questionable to discuss the relationship between Internet and residents' fitness behaviors only by using “media mobilization” and “media suppression.” Secondly, as most studies have been conducted only to assess the positive or negative effects of the Internet on physical fitness, the relationship between the two has long been discussed only in terms of “mobilization” and “suppression,” with less research on the more profound mechanisms. Since the outbreak of COVID-19 epidemic, several studies have theoretically examined the role of social risk (11), social anxiety (24), and social interaction (10) in the process of the Internet influencing the physical fitness behavior of the residents, but these studies have not sufficiently tested the theory.

With the regular prevention and control of the epidemic, physical fitness is still an essential method in protecting the physical and psychological health of the residents. To examine the functional mechanism and relationship of the Internet in affecting the residents is of great theoretical value and practical meaning. Therefore, this study concentrates on residents' physical fitness behavior and utilizes the micro-panel data of China Family Panel Studies (CFPS) in 2014, 2016, 2018, and 2020 to analyze the influence and functional mechanisms of the Internet. The purpose of this study was to: (1) examine whether there exists a relationship between the Internet and residents' behaviors of physical fitness; (2) explore what the specific relationship is; (3) find the functional mechanism through which the Internet influence residents' physical fitness behavior.

## Literature review

### Social compensation and time displacement

Currently, the results of empirical studies on the impact of the Internet on the residents' physical fitness behaviors vary significantly. Some studies concluded that the overall effect of the Internet on physical fitness is positive (13, 16); while others indicated the opposite (25), and some studies suggested that there was no significant relationship between the two (18). Theoretically speaking, the influence of the Internet on residents' physical fitness has effects of both social compensation and time displacement. Scholars holding the view of social compensation argue that, as a critical way to obtain information resources in modern society, the Internet has more dominance in shaping the sports environment, acquiring sports knowledge, skills, spectating and other resources, which to a certain extent compensates for the dilemma of insufficient sports resources possessed by the underprivileged and assists in improving residents' sports and fitness awareness, concepts and behaviors (26). Media effects theory promotes that the media sports integrated by the Internet and sports has both social and individual communication effects: at the social level, sports media can generate the effects of sports environment cognition, sports fitness value formation and maintenance, and sports social behavior demonstration; at the individual level, sports media can cause changes in residents' sports cognition, sports attitudes, and sports behaviors, and this effect continuously accumulates, deepens and expands. However, the theory also reveals that there exists no direct relationship among individual cognition, attitude and behavior (27). In other words, the Internet medium can change residents' perceptions and attitudes toward physical fitness, without necessarily affecting their fitness behaviors. Consequently, media effects theory can account for the rapid development of sports media and the widespread use of smart sports devices in China without changing the physical fitness behavior of the residents, especially the youth group. Internet may be a positive, but not decisive, factor influencing residents' fitness behavior.

As an effective way of social interaction, the Internet is an alternative form of social capital acquisition (28, 29), indirectly influencing residents' physical fitness behavior. Social capital theory suggests that resources embedded in interpersonal social relationship networks play an important role in the achievement of individual behavioral goals, and that interpersonal interactions in real society are the principal way to obtain social capital, while online social networking, although different from the real social interactions, can also establish interpersonal relationships and may even obtain more superior and extensive social capital accumulation (30). As many types of research reveals, social capital accumulated from interactions in real society has a positive effect on residents' physical fitness behaviors (31, 32). However, with the continuous development of Internet information technology, depending on Internet social media to obtain social capital is gradually becoming a principal way to promote residents' physical fitness levels. Bian and Lei concluded that online socialization expands the characteristic heterogeneity, hierarchical breadth, and status of social interaction groups, thus contributing to the consolidation and expansion of both in-group and out-group social capital (33). They proposed the concept of online epidemic prevention social capital and verified the capital acquired by residents through the Internet medium during the epidemic had a significant positive effect on residents' physical fitness (34). Based on big data from 7 countries worldwide, Lu used methods of computational sociology to further empirically confirm the effectiveness of online social networking in enhancing the online fitness behavior of the residents, which has a positive effect on epidemic prevention and the health of the residents (10). In addition, in a study based on data from the 2012 China General Social Survey, Wang suggested that online media can not only expand the network size of social relationships, but also provide individuals with more social support and emotional comfort, which in turn is conducive to cultivating residents' physical exercise habits (35). Therefore, residents' behavior of Internet use may have exceeded the single function of searching for sports and fitness information and transferring resources, and become a critical tool for acquiring social capital, thus indirectly influencing residents' sports participation.

Scholars who support the time displacement effect claim that time spent on Internet crowds out time for physical fitness with negative effects on residents' fitness behavior. The time displacement effect hypothesis states that the total amount of disposable time is limited and there is a zero-sum relationship between the time occupied by various behaviors (36). On this basis, Robinson propose the functional equivalence hypothesis, which suggests that media with the same function or providing the same satisfaction are most likely to be displaced by new media (37). With the universalization and development of Internet technology, network media have a significant time displacement effect on mass media such as TV, radio, newspapers and books (38, 39), but the effect on individual behavior is associated with the type of daily activities (37). Some studies suggested that the Internet medium has a time displacement effect on children and adolescents' physical fitness behavior (40, 41). What's more, the effect of time displacement was also examined in research of the relationship between network media and social capital (42, 43). For instance, Putnam suggested that in1970's, hidden behind the phenomenon of “bowling alone” in the US is the fact that digital media have occupied people's public living space, resulting in less time for human interaction and social capital loss (44). However, it is still uncertain whether the time displacement effect of the Internet weakens the impact of social capital on physical fitness.

Above all, the Internet has a dual effect on residents' physical fitness behavior. In terms of the time displacement effect, there may be a time competition relationship between Internet and physical fitness, i.e., the increase in the duration of Internet use will crowd out the time of physical fitness. However, in regard to the social compensation effect, the Internet has a positive impact on residents' physical fitness behavior, in other words, the social compensation effect of the Internet media is reflected as a tool both for transferring sports information resources and obtaining social capital. Existing studies still have some limitations: firstly, studies mainly use cross-sectional data, which makes it difficult to examine the causal relationship between the Internet and physical fitness behavior; secondly, previous studies have focused on only one dimension of physical fitness frequency, time, and effectiveness, and rare studies have examined the effect of the Internet from multiple dimensions simultaneously; thirdly, different from the previous circumstances, the social interactions of Chinese residents are subject to the development of the epidemic under the regular prevention and control, and the existing studies have not examined and responded to the effects of social capital mechanisms. In addition, another significant reason for the influence of the Internet on physical fitness behavior is the transformation of residents' health anxiety and perceptions during the epidemic.

### Health risk perception

With the progress of industrialization and urbanization in China, the characteristics of a “risk society” are emerging, and various “social risks” such as environmental pollution, food safety, and infectious diseases are reinforced by the Internet increasing the perceived health risks of the residents (45). In exploring the relationship between media use and individual behavior from the perspective of risk communication, scholars have proposed theories like “social amplification of risk” (46), “protection motivation theory” (47), and “media credibility” (48). However, the basic analytical framework of these theories is based on the logic of “information-perception-behavior.” Thus, “perception” is an essential link between “information” and “behavior,” in other words, health perception may be another pathway through which the Internet influence residents' physical fitness behavior.

To begin with, there is either a weakening or strengthening relationship between the Internet and health perceptions. According to studies on the weakening relationship, in contrast to traditional societies, people's perception of modern society is mainly obtained by the media, and the social amplification effect of the media on risk increases the level of social risk perception (49), and also the level of health risk perception (50). At the beginning of the outbreak, physical isolation allowed the Internet media to become an effective way for residents to follow the development of the epidemic, but the public and even experts had little knowledge and measures to effectively prevent and control the virus. The negative information that was amplified during the dissemination of the Internet media caused excessive public panic and health concerns, and the negative emotions of anxiety significantly increased the level of risk perception of residents' physical and mental health status, and even resulted in irrational crowds behaviors such as the “crazy purchasing of food and vegetables” and the “rush for Shuanghuanglian and Banlangen.” Despite the fact that official media propaganda on epidemic prevention and control knowledge and measures has reduced the risk of social amplification effect of the Internet (48). However, once the new epidemic rebounded in a certain area, the social amplification effect of online media would again be prominent and influence the level of risk perception and health behavior of the residents. Research on strengthening relationships argues that Internet will facilitate residents' health perceptions. Compared to the social amplification theory of risk from the communicator's perspective, the “use and satisfaction” theory considers the exposure or use of media from the audience's position as a deliberate action based on one's own needs to meet specific individual needs (51). In a study on health perceptions of the elderly, He and Yuan found that the use of Internet media can satisfy the need for emotional communication and thus have a significant positive impact on the physical and psychological health perceptions of the elderly (52). Wang identified that the use of online media can reduce stress and tension from daily issues, help individuals access more health information and medical resources, and thus improve their perception of health (35). Despite the fact that some studies have questioned this view (53), it is undeniable that media socialization was an effective complement to daily social activities during the epidemic, which contributed to a decrease in loneliness and emotional enrichment among the elderly population (54, 55).

Moreover, the impact of health risk perception on physical fitness has also been focused on and discussed by scholars. The behavioral theory holds that health perceptions, attitudes, motivation, self-efficacy, and self-identity are all major factors influencing physical activity and can predict, to varying degrees, individual physical fitness levels (56). The Health Belief Model proposes that individuals have the confidence and ability to change undesirable behaviors when they are in fear of the current malpractice and are convinced that they will derive more benefit (56). With the arrival of the “risk society” and the fast-paced lifestyle, people are increasingly aware of the importance and urgency of health, and the perception of health status has become an crucial subjective factor influencing individual sports participation in the “post-epidemic” era (11, 57). Huang et al. theoretically explores the logic between the social mindsets triggered by Internet media during the epidemic, such as public panic and anxiety, and the action of physical fitness, suggesting that the health concerns of the public and the introspection of their own behavior are rational expressions of the social action of physical fitness to resolve social anxiety (24). It is suggested that health risk perceptions significantly promote physical activity among adolescents (58) and play an important mediating role in the relationship between self-efficacy and physical activity (59). Based on the above analysis, this study consider that the health risk perception may be another important mechanism through which the Internet influence physical fitness behavior.

## Materials and methods

### Data sources

The research data in this paper comes from the China Family Panel Studies (CFPS) database, conducted by Institute of Social Science Survey, Peking University and the survey was conducted every 2 years since 2010. In the four surveys from 2014 to 2020, CFPS collected data from three levels, individual, household, and community, among which the data of respondents' physical fitness, Internet use, social participation, health status evaluation, and demographic characteristics provided precious information for this study to explore the causal analysis and mechanism of Internet use and physical fitness. Unfortunately, only individual-level data of CFPS2020 are available, which cannot be effectively matched with household-level data in other surveys, and the questionnaires on Internet use and physical fitness behavior are designed with a different format from other surveys. Hence, in order to reduce the measurement error and ensure the authenticity and reliability of the results, this study selected the data of 23,989 samples successfully followed in all three surveys of CFPS from 2014 to 2018 to explore the causal effect of the relationship between the Internet and physical fitness; meanwhile, the data of 24,687 samples in CFPS 2020 to examine the mechanism of the Internet's effect on residents' physical fitness behavior. The characteristic of the selected data is consistent with the assumptions of cross lagged models, which are contemporaneity, smoothness, and period effects. First, the CFPS data used in this paper contain data for three periods, 2014, 2016, and 2018. Second, each variable is measured at the same scale and occurs within the same period. Cross lagged models are an important method of causal inference and are widely used in panel data analysis. Michael W. Kearney describes the principles for this method of causal inference in detail in his article Cross Lagged Panel Analysis.

### Variable selection

#### Dependent variable

The current academic measurement of physical fitness includes three main dimensions: frequency, time, and effectiveness, and the selected dependent variables are the frequency, duration, and effectiveness of residents' physical fitness. The question in the questionnaire of CFPS2014, 2016, and 2018 is “How many times have you participated in physical fitness in the past week?” and “How long did you participate in physical activity in total?”; the range of answers for the frequency of fitness is 0 to 21 times/week, and the range of answers for the time of fitness is 0.1 to 105 h/week. However, there were no results on fitness effectiveness in the survey. The CFPS 2020 survey inquired about the frequency, time, and effectiveness of physical fitness in the past year. The answer to the question of frequency is “(1) <1 time per month on average,” “(2) More than 1 time per month on average, but <1 time per week,” “(3) 1–2 times per week on average,” “(4) times per week on average,” “(5) times per week and above on average,” “(6) 1 time per day on average,” “(7) Twice a day and above on average,” and “(8) Never participate.” This study re-codes and re-assigns the above answers, options 1 and 8 are assigned as 0 “Never participate,” options 2 and 3 are assigned as 1 “Low,” options 4 and 5 are assigned as 2 “Middle,” and options 6 and 7 are assigned as 3 “High.” The duration of physical fitness ranges from 1 to 300 min per day. The answer of effectiveness is “breathing and heart rate do not change much,” “increased breathing, heart rate, slight sweating,” and “Shortness of breath, significantly faster heartbeat, more sweating,” which are coded and assigned as (1) “average,” (2) “good,” and (3) “excellent,” respectively.

#### Independent variable

The independent variable of this study is whether to use the Internet, including mobile Internet access and computer Internet access. A respondent is considered to be using the Internet medium if he or she uses any of the methods to access the Internet and is assigned as 1. If not, assigned as 0. The independent variable is defined as “Internet use.” Meanwhile, CFPS 2014–2018 investigated residents' average time spent online per week, while CFPS 2020 surveyed residents' time spent online per day. Therefore, this study standardizes the unit of Internet access time as “hours per week” and takes its logarithmic value, which is defined as Internet duration.

#### Mediator variable

The selected mediator variables are social capital and health risk perception, as is showed in Table 1. Participation in social organizations is an important indicator of an individual's social capital, which is measured by the question “which of the following organizations do you currently participate in?”. A total of 5 social organizations were selected, and respondents will be assigned as 1 if they were a member of any of these organizations, and 0 if they were not. This study refers to Sherbourne's measure of assessing health risk perception and selects the question “Do you think your health is excellent, very good, good, not bad, or poor” (60). Also, eight questions from the Center for Epidemiological Studies-Depression (CES-D) scale are selected and summed to measure the perception of psychological health risk. The higher the two variables indicate a higher level of health risk perception. It should be clarified that the CFPS 2014 does not include the CES-D depression scale.

**Table 1:** Descriptive statistical results of variables.

**Categories**	**Variable**	**2014**	**2016**	**2018**	**2020**
		**(*****N*** = **22,471)**	**(*****N*** = **22,540)**	**(*****N*** = **21,707)**	**(*****N*** = **23,872)**
Dependent variable	Frequency	1.85 (2.93)	2.18 (3.08)	2.68 (3.29)	
	Never participate = 0				63.64
	Low = 1				15.03
	Middle = 2				7.45
	High = 3				15.6
	Time	1.58 (0.98)	1.67 (0.98)	1.70 (0.98)	3.86 (0.65)
	Effectiveness				
	Average = 1				20.21
	good=2				48.89
	Excellent = 3				30.9
Independent variable	Internet use				
	Not use = 0	71.34	61.66	52.31	34.01
	Use=1	28.66	38.34	47.69	65.99
	Internet duration	2.03 (0.99)	2.09 (1.07)	2.16 (1.07)	4.81 (1.33)
Mediator variable	Social capital (participate in organization = 1)	14.99	26.99	22.16	26.28
	Physical Health risk perception (1–4)	2.98 (1.25)	3.11 (1.23)	3.13 (1.24)	2.81 (1.20)
	Psychological health risk perception (8–32)	—	13.20 (3.91)	13.56 (4.05)	13.44 (4.03)
Controlled variables	Age (9–100)	46.29 (16.38)	48.39 (16.32)	50.30 (16.38)	42.51 (18.29)
	Gender (male = 1)	49.4	49.41	49.37	50.42
	Marital status (married = 1)	80.82	81.47	76.11	70.54
	Years of education (0–24)	7.28 (4.69)	7.35 (4.80)	7.52 (4.91)	8.61 (4.58)
	Household income per capita	9.04 (1.18)	9.39 (0.99)	9.56 (1.20)	—
	Urban = 1	44.46	45.84	48.11	49.82
	BMI (1–4)	2.28 (0.71)	2.32 (0.72)	2.36 (0.72)	2.31 (0.78)

#### Controlled variables

Referring to the previous literature, this study selects control variables including age, gender, marital status, years of education, household income per capita, urban-rural residence, and BMI, among which the per capita household income is logarithmically processed. The results of the coding value and descriptive statistics of each variable are shown in Table 1.

#### Model selection

Table 1 shows the changing trends in Internet use, time spent and physical fitness behavior of Chinese residents. Compared with 2014, the percentage of Internet use among residents in 2020 has increased by 18.27% and the average weekly usage time has risen over 50%. The frequency and time of physical fitness among residents also show an upward trend from 2014 to 2018, but the proportion of residents who do not participate in physical fitness is close to 66% in 2020, and < 15.6% insist on daily fitness. There also exists slight fluctuations in the social capital and health perceptions possessed by residents around the time after the outbreak of the COVID-19 epidemic, but the overall trend is on the rise. In addition, it is noteworthy that variables that shift over time are not entirely individual and may be influenced by changes over time in sample size or other factors. Under such circumstances, conventional mixed regression models may not accurately estimate the relationship between Internet and physical fitness behavior, resulting in incorrect research findings. Therefore, this study firstly applies the Time Fixed Effects Model to address the problem of omitted variables that vary with individuals and control for the effect of confounding structural variables that vary with year. Then, this study utilizes the Cross Lagged Panel Model to examine the causal relationship between the Internet and physical fitness behavior by maximum likelihood estimation method. Finally, this study verifies the mediating role of social capital and health perceptions in the relationship between the Internet and physical fitness behavior through the KHB model.

## Results

### Time fixed effects model

To examine the effects and duration of Internet use on the frequency and time of physical fitness residents, respectively, six models were constructed and the effects of time-varying factors and confounding bias caused by individual factors were controlled for by incorporating time dummy variables and relevant individual factors for the survey years, and the regression results are shown in Table 2. In terms of fitness frequency, the results of model 1 indicate that Internet use can positively influence fitness frequency compared with residents who do not use the Internet; the results of models 2 and 3 show that there is no linear but non-linear relationship between the Internet duration and fitness frequency, and fitness frequency presents a trend of first decreasing and then increasing with the rise in using duration. This suggests that Internet use has a social compensation effect on residents' physical fitness behavior. In regard to fitness time, the results of model 4 demonstrate that Internet use does not significantly increase fitness time compared to residents without using the Internet; however, the results of model 5 show that Internet use duration has a significant positive correlation with fitness time, yet a non-linear relationship between the two is not found in model 6, suggesting that the time displacement effect of Internet use on residents' physical fitness behavior is not verified. The above results reveal the relationship between residents' Internet use and physical fitness behavior and the differences in their effects. However, the relationship may be influenced by unobserved omitted variables, such as spatial differences in Internet penetration in China (60, 61), and thus no accurate judgment can be made about the causal relationship.

**Table 2:** The influence of Internet on the frequency and time of residents' physical fitness.

**Variable**	**Fitness frequency**			**Fitness time**		
	**Model 1**	**Model 2**	**Model 3**	**Model 4**	**Model 5**	**Model 6**
Internet use	0.278^***^			0.007		
Internet duration		−0.004	−0.098^*^		0.055^***^	0.029
Internet duration squared			0.026^*^			0.008
Social capital	0.270^***^	0.122^**^	0.122^**^	0.004	−0.03	−0.029
Physical health risk perception	−0.058^***^	−0.045^*^	−0.045^*^	−0.016^*^	0.009	0.009
Psychological Health risk perception	0.001	−0.022^***^	−0.022^***^	−0.006^**^	−0.008^*^	−0.008^*^
Age	−0.048	−0.125^**^	−0.124^**^	0.019	0.029	0.03
Age squared	0.001^***^	0.003^***^	0.003^***^	0	0	0
Gender (male = 1)	−0.069	−0.376	−0.367	−0.075	1.470^***^	1.476^***^
Marital status (Married = 1)	−0.160^*^	−0.267^**^	−0.264^**^	−0.089^*^	−0.12	−0.117
Years of education	−0.063^***^	−0.025	−0.026	−0.021^*^	−0.027^*^	−0.027^*^
Household income per capita	−0.026	0.01	0.01	0.022^**^	0.026	0.026
Urban = 1	0.093	0.091	0.093	0.031	0.049	0.049
BMI	0.037	−0.011	−0.013	0.019	−0.011	−0.012
2016	0.148^*^	0.411^***^	0.406^***^	0.145^***^	0.126	0.125
2018	0.494^***^	0.586^***^	0.579^***^	0.162^**^	0.106	0.103
Intercept	1.956	3.086^*^	3.112^*^	1.096	−0.379	−0.386
R2	0.033^***^	0.100^***^	0.110^***^	0.048^***^	0.018^***^	0.018^***^
*N*	66,708	25,443	25,443	27,934	12,746	12,746

Standard errors have been omitted for simplicity of presentation.

* p < 0.1,

** p < 0.05,

*** p < 0.01.

### Cross lagged panel model

**Figure 1:**
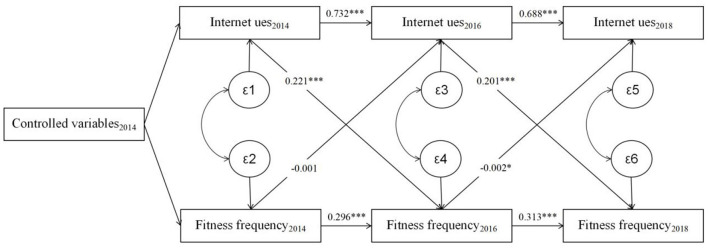
The estimation of the cross lagged panel model of the internet use and physical fitness frequency.

**Figure 2:**
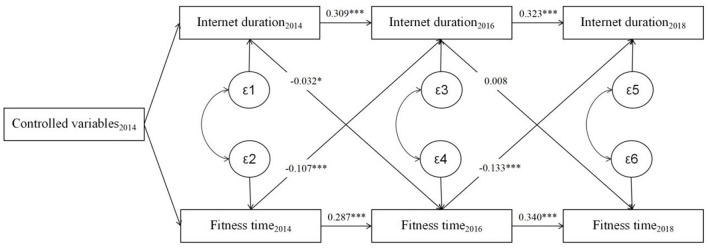
The estimation of the cross lagged panel model of the internet duration and physical fitness time.

This study employs a cross lagged panel model to further estimates the causal relationship between Internet use and physical fitness behavior. The model is currently an important method widely used to explore causality. Based on panel data of two or more periods, it estimates the cross-period effects of variables on the basis of the predicted contemporaneous correlations between variables and self-correlation of variables (i.e., controlling for contemporaneous effects and time-period variance), in other words, it determines the causal effects between the independent variables by estimating the significance of the effect parameter β1 of the dependent variable in the previous period on the dependent variable in the latter period and the effect parameter β2 of the dependent variable in the previous period on the independent variable in the latter period (62). The assumptions of the model include: each measured variable occurs at the same time period; the relationship between variables remains consistent over time (especially for three and more periods), and the length of the period should not be set too long or too short, etc. According to the assumptions of the model, the variables of “Internet use,” “Internet duration” and fitness “frequency” and “time” with the same measurement scale in the survey data of 2014, 2016, and 2018 were selected for this study, and other characteristic variables that could cause confounding bias within the same period were controlled. After accepting the autocorrelation of inter- and intra-group variables across time, the estimated results of the causal relationship between residents' Internet use and physical fitness behavior are shown in the Figures 1, 2.

The analysis results of Internet use and fitness frequency are presented in Figure 1. At first, both Internet use and fitness frequency show significant autocorrelations, with stronger positive correlations between Internet use than fitness frequency in the two consecutive periods. Secondly, the results of the model analysis constructed with three temporal points of Internet use and fitness frequency reveal that the first and second measure of Internet use significantly predict the second and the third measure of fitness frequency (β_t − 1_ = 0.221, *p* < 0.001; β_t − 2_ = 0.201, *p* < 0.001). While the first measure of fitness frequency does not significantly predict the second measure of Internet use (β_t − 1_ = −0.001, *p* > 0.1), and the impact of the second measure of fitness frequency on the third measure of Internet use was only negative significant at the 0.1 level (β_t − 2_ = −0.002, *p* < 0.1). This is consistent with the results of the previous analysis, thereby further suggesting that Internet use is responsible for the increase in the frequency of fitness among residents.

The results of the analysis of Internet duration and fitness time are illustrated in Figure 2. Similar to fitness frequency, fitness time and Internet duration indicate a significant autocorrelation. In terms of causal direction, the first measure of Internet duration negatively predicts the second fitness time at a significant level of 0.1 (β_t − 1_ = −0.032, *p* < 0.1), while the second measure of Internet duration has a non-significant effect on the third measure of fitness time (β_t − 2_ = 0.008, *p* > 0.1). However, the first and second measures of fitness time significantly and negatively predict the second and third measures of Internet duration (β_t − 1_ = −0.107, *p* < 0.001; β_t − 2_ = −0.133, *p* < 0.001), suggesting that the decrease in fitness time is responsible for the increase in Internet duration; in other words, the increase in physical fitness time significantly reduces Internet duration.

The results of the cross lagged model verified the causal relationship between Internet and physical fitness behavior, thus further indicating the existence of social compensation and time displacement effect of Internet on physical fitness behavior. Firstly, there is a rivalry between fitness time and Internet duration, where an increase in the former reduces the latter. Secondly, compared to residents who do not use Internet, users' social compensation effects have a positive impact on their physical fitness behavior. This study will further analyze the mechanism of the social compensation effect brought by the Internet on physical fitness behavior.

### Functional mechanism of Internet use in influencing residents' physical fitness behavior

Based on the previous theoretical discussion and the CFPS2020 cross-sectional data, this study verified the role of social capital and health risk perceptions in influencing the physical fitness behavior of residents through the Internet in the context of regular epidemic prevention and control. Since fitness frequency and effectiveness are ordered categorical variables in the CFPS2020, this paper first applied Ologit estimation method to construct a multivariate nested model to examine the relationship between Internet use, social capital, health risk perception and physical fitness behavior, and then conducted a mediating effect test by KHB analysis. The advantage of KHB analysis is that it can be adopted in mediating effects analysis of nonlinear logistic regression models to compare the estimated coefficients of the same sample of nested models, so as to effectively estimate and test the mediating effects (63).

As indicated in Table 3, Internet use can also significantly increase residents' fitness frequency and effectiveness after controlling for the same individual characteristic variables in the previous section. Specifically, the frequency and effectiveness of fitness are 91.3 and 17.1% higher for residents who use the Internet than for non-users. After adding social capital and health risk perceptions to models 1 and 5, respectively, the results of models 2 and 6 indicate that social capital has a significant positive effect on the increase of fitness frequency of residents, but not on the effectiveness. Models 3 and 7 suggest that the lower the perception of psychological health risks, the higher the frequency of fitness; the higher the perception of physical and psychological health risks, the better the fitness effectiveness is. As can be seen from Models 4 and 8, when both social capital and health risk perception variables are included, residents with higher social capital and psychological health risk perceptions have higher fitness frequency. Higher the physical and psychological health risk perceptions result in better fitness, but social capital has no significant effect on the fitness effect. With the use of Internet, the odds ratio of fitness frequency among residents decreased by 1.9%, but there is an increase of 1.7% in the odds ratio of fitness effectiveness. The result suggests a possible mediating effect of social capital and health risk perceptions between Internet use and residents' fitness behavior, which will be further examined by this study through KHB analysis method.

**Table 3:** The influence of the Internet on the frequency and fitness effect of residents' physical fitness under regular epidemic prevention and control.

**Variable**	**Fitness frequency**				**Fitness effectiveness**			
	**Model 1**	**Model 2**	**Model 3**	**Model 4**	**Model 5**	**Model 6**	**Model 7**	**Model 8**
Internet use	1.913^***^	1.910^***^	1.896^***^	1.894^***^	1.171^**^	1.171^**^	1.188^**^	1.188^**^
Social capital		1.304^***^		1.332^***^		0.98		0.985
Health risk perception								
Physical Health risk perception			0.987	0.988			1.065^***^	1.065^***^
Psychological health risk perception			0.894^***^	0.895^***^			1.249^***^	1.249^***^
Age	0.923^***^	0.921^***^	0.928^***^	0.927^***^	1.007	1.007	0.990	0.991
Age squared	1.001^***^	1.001^***^	1.001^***^	1.001^***^	1.000^***^	1.000^***^	1.000^**^	1.000^**^
Gender (male = 1)	1.071^**^	1.076^***^	1.053^*^	1.059^**^	1.292^***^	1.292^***^	1.341^***^	1.341^***^
Marital status (Married = 1)	0.685^***^	0.713^***^	0.664^***^	0.690^***^	0.945	0.942	1.008	1.006
Years of education	1.570^***^	1.494^***^	1.552^***^	1.477^***^	0.946^*^	0.949^*^	0.977	0.979
Urban = 1	1.740^***^	1.737^***^	1.725^***^	1.722^***^	1.094^**^	1.094^**^	1.109^**^	1.109^**^
BMI	1.065^***^	1.066^***^	1.063^***^	1.063^***^	1.225^***^	1.225^***^	1.229^***^	1.229^***^
R2	0.055^***^	0.057^***^	0.057^***^	0.058^***^	0.033^***^	0.033^***^	0.041^***^	0.041^***^
*N*	23,872	23,872	23,872	23,872	9,073	9,207	9,207	9,207

The coefficient estimate is Exp.

Standard errors have been omitted for simplicity of presentation.

* p < 0.1,

** p < 0.05,

*** p < 0.01.

The results of the KHB test for the mediating variables and their decomposition effects are listed in Table 4. Social capital and health risk perceptions exert mediating effects in the relationship between Internet use and fitness frequency, but no mediating effect is found in the effect of Internet use on fitness effectiveness. After simultaneously including the three mediating variables of social capital, physical and psychological health risk perceptions in the main effects model, the direct effect of Internet use on fitness frequency is significantly reduced by 0.0159, resulting in indirect effects of 0.0046, 0.0003 and 0.0106, at the individual contribution rate of 30.56, 0.22, and 69.27%, respectively. The contribution rate of psychological health risk perception is higher than that of social capital, and the rate of physical health risk perception is the lowest. After adding the three mediating variables separately in the main effects model, only the mediating effect of psychological health risk perception is significant with a separate contribution rate of 2.16%, while the mediating effect of the rest was not significant. The results indicate that the social compensation effect of Internet use on residents' fitness behavior is mainly reflected in the increase of social capital and the decrease of health risk perceptions, while under the regular prevention and control of the epidemic, residents' psychological health risk perceptions are a critical mechanism for media use to affect fitness behavior.

**Table 4:** Mediating effects test based on the KHB.

**Variable**	**Fitness frequency**			**Fitness effectiveness**		
	**Social capital**	**Physical health risk perception**	**Psychological health risk perception**	**Social capital**	**Physical health risk perception**	**Psychological health risk perception**
Total effects	0.649^***^	0.649^***^	0.649^***^	0.171^**^	0.171^**^	0.171^**^
Direct effects	0.644^***^	0.649^***^	0.635^***^	0.158^**^	0.175^**^	0.189^***^
Indirect effects	0.005	0	0.014^**^	0	−0.004	−0.018
Separate contribution rate	—	—	2.16%	—	—	—
Individual contribution effect	0.005	0.0003	0.0106	—	—	—
Individual contribution rate	30.56%	0.22%	69.21%	—	—	—

Standard errors have been omitted for simplicity of presentation.

** p < 0.05,

*** p < 0.01.

## Discussion

In this study, we applied the first 3 years of CFPS data to explore the casual effect of Internet use and residents' physical fitness. With the data from CFPS2020, we further explored the functional mechanism of the Internet on residents' physical fitness behavior under the regular prevention and control of the COVID-19 epidemic from two dimensions: social capital and health risk perception. We obtained three valuable conclusions as follows: First, there is a significant correlation between the Internet and physical fitness behavior. Second, the Internet use is the cause for the increase in fitness frequency, and there is a rival relationship between Internet duration and fitness time. Third, under regular prevention and control of the COVID-19 epidemic, social capital and health risk perceptions are the functional mechanisms of the Internet influencing fitness behavior, and the mediating effect of psychological health risk perceptions is higher than that of social capital.

During 2014–2018, Chinese residents' Internet use and physical fitness behavior both showed an increasing trend, with the proportion of people using mobile devices such as cell phones and computers to access the Internet increasing from 28.66 to 65.99%, and the duration of usage increasing from 11.7 to 25 h per week. Physical fitness frequency and time increased from 1.8 times/week and 7.8 hours/week in 2014 to 2.7 times/week and 9.0 h/week in 2018, respectively, but the proportion of people who participated in 1 time or more decreased in 2020, and fitness time maintained at 1 h/time. The results of the fixed effects model confirms the “media mobilization theory” (14, 15) proposed in previous studies. Both Internet use and duration have a significant positive effect on fitness behavior, but there is no significant difference in the time spent on fitness whether or not they use Internet; nor do increase the time spent on Internet significantly raise the frequency of fitness. Thus, it can be seen that the effect of the Internet on fitness behaviors may vary in findings due to differences in concept measurement, which may partially explain the “media suppression theory” that has been upheld by previous empirical studies (16, 17).

In contrast to previous studies, the CFPS data used in this study allowed us to explore the causal relationship between the Internet and fitness behaviors. The results of the cross-lagged panel model reveal that the use of Internet significantly and positively predicts the frequency of fitness; while the Internet duration negatively predicts the fitness time and vice versa. This demonstrates that the influence of Internet use on fitness behavior has both a “time displacement effect” and a “social compensation effect.” In terms of fitness time, as the total amount of time is limited, increasing the time spent on Internet use will inevitably be at the cost of decreasing the time spent on fitness, and in turn, increasing the time spent on fitness will also certainly reduce the time spent on Internet use, therefore, this study supports the “time displacement effect hypothesis” of the Internet. Internet media also has an important “social compensation effect” on fitness frequency. Previous studies have suggested that the social compensation mechanism of the Internet is mainly reflected in its significant role in shaping the social environment of sports, accessing fitness information and resources, and altering fitness attitudes and motivation (19, 20). The disadvantaged can use the Internet to compensate for the insufficient possession of social sports resources and thus promote their fitness behavior (26). However, this study suggests that with the extensive use of Internet information technology, residents' Internet use behavior may have exceeded the single function of searching or delivering sports information, and may be more likely to become an effective tool for social interaction and health risk perception, further shaping residents' fitness behavior.

Although it has been shown that epidemic prevention social capital formed through the Internet during the physical isolation of the COVID-19 epidemic had a significant effect on physical fitness (9, 10, 34). This study show that when the mediating effects of social capital and health risk perceptions were examined simultaneously, the contribution of social capital in the total mediating effect is 30.56%, which is lower than the 69.27% contribution of psychological health risk perceptions. When the mediating effects of social capital and health risk perceptions are examined separately, the mediating effect of social capital is not significant, while the mediating effect of psychological risk perceptions is, but only accounted for 2.16% of the total. This study suggests that online and face-to-face social interaction are two forms of social capital accumulation, and that the Internet is an effective way for residents to interact with the outside world and to achieve social capital during the epidemic isolation period, however, as for the regular prevention and control of the epidemic, real social interaction may still be the main way to maintain and develop social capital. Therefore, due to the impact of the regular prevention and control of COVID-19 epidemic, the mediating role of social capital in influencing fitness behavior through online media is diminished. The mediating effect of psychological health risk perceptions is stronger than that of physical health risk perceptions, and although the separate contribution of physical health risk perceptions is not significant, the mediating effect of psychological health risk perceptions is. On one hand, the health threat of the epidemic is intensified by the “amplification effect of the risk society” of the Internet, which in turn motivates residents to pay more attention to healthy lifestyles, especially physical fitness; on the other hand, the psychological impacts of the epidemic are more extensive, longer-lasting, and more easily perceived than the physical ones. With the virus becoming less severe and the effective implementation of China's epidemic prevention and control policy, the daily life of residents gradually returns to normal and the perception of physical health risk will gradually decrease, but the perception of psychological health risk could hardly diminish in a short period of time.

There are some limitations to be interpreted. First, due to data limitations, we could only measure the concept of “Internet” in two dimensions: “Internet use” and “Internet duration,” which would generalize the findings of this study. Second, the sample of this study included three social groups adolescents, middle age, and the elderly. Thus, this study examined the causal relationship and mechanism of action between online media and residents' fitness behavior from a general perspective without considering the issue of age heterogeneity. For this reason, the total mediating effect of social capital and health risk perception was low when the mediating effect was tested.

Despite these limitations, our study revealed the causal relationship and functional mechanism between the Internet and physical fitness in terms of theoretical derivation and data validation. Therefore, it also provides practical recommendations for future work. First, We should accelerate the deep integration of “Internet + fitness” and optimize the function of sports media in promoting scientific fitness knowledge, skills and methods. Sports media is an achievement of integrating and developing the Internet and sports and has played an essential role in sports communication. However, the current content of sports media communication is based on the broadcast of major international and domestic sports events. Very little content is disseminated for scientific sports and fitness knowledge, skills and methods. Although we can search for the fitness content we need in online media, it lacks authority and may have a negative impact on residents' physical fitness. Second, we need to reasonably allocate time for Internet use, avoid the time displacement effect, promote the effect of social capital accumulation and avoid the effect of health risk perception. With the rapid growth of global Internet penetration, the online medium has become an essential way of online services such as online shopping, payment, education, medical care, and social interaction in developed and developing countries. However, the total amount of individuals' time is limited, and prolonged physical behavior on the online medium shortens the time spent on physical fitness. Compared to the time displacement effect of the Internet, the social capital accumulation effect facilitates residents' fitness behavior. Although this study found that the mediating effect of risky health perceptions was greater than that of social capital in Internet influences on physical fitness during the particular period of COVID-19, as the impact of the epidemic continues to decrease, social capital could still be an essential functional mechanism for the Internet to influence physical fitness behavior.

## Conclusion

This study explored the causal relationship and functioning mechanism of Internet and physical fitness behavior. We found that there is a significant correlation between the Internet and physical fitness behavior, the Internet use is the cause for the increase in fitness frequency, and there is a rival relationship between Internet duration and fitness time. Moreover, under regular prevention and control of the COVID-19 epidemic, social capital and health risk perceptions are the functional mechanisms of the Internet influencing fitness behavior, and the mediating effect of psychological health risk perceptions is higher than that of social capital. At the same time, we also observed a relatively moderate effect of Internet on fitness behavior. Therefore, how to further create an intelligent, informative and digital sports public service system *via* enriching and optimizing sports medium, and facilitate the Internet to better serve the residents' physical fitness is a theoretical and practical topic that should be focused in the future.

## Data availability statement

The original contributions presented in the study are included in the article/supplementary material, further inquiries can be directed to the corresponding author/s.

## Ethics statement

The studies involving human participants were reviewed and approved by Ethics Committee of the School of Xi'an Jiaotong University. The patients/participants provided their written informed consent to participate in this study.

## Author contributions

LZ: conceptualization. CL and LZ: data collection, writing–original draft, and writing–review and editing. CY: methodology and supervision. ZL: writing–review and editing. YZ: supervision. All authors have read and agreed to the published version of the manuscript.
